# Wide-field imaging with smartphone based fundus camera: grading of severity of diabetic retinopathy and locating peripheral lesions in diabetic retinopathy

**DOI:** 10.1038/s41433-024-02928-2

**Published:** 2024-01-31

**Authors:** Ramachandran Rajalakshmi, Rajah Mohammed, Kalaivani Vengatesan, Thyparambil Aravindakshan PramodKumar, Ulagamathesan Venkatesan, Manoharan Usha, Subramanian Arulmalar, Vijayaraghavan Prathiba, Viswanathan Mohan

**Affiliations:** 1 Department of Ophthalmology, Dr. Mohan’s Diabetes Specialities Centre and Madras Diabetes Research Foundation, Chennai, India; 2 Department of Research Operations and Diabetes Complications, Madras Diabetes Research Foundation, Chennai, India; 3 Department of Biostatistics and Data Management, Madras Diabetes Research Foundation, Chennai, India; 4 Department of Diabetology, Dr. Mohan’s Diabetes Specialities Centre and Madras Diabetes Research Foundation, Chennai, India

**Keywords:** Retinal diseases, Outcomes research

## Abstract

**Aim:**

To assess the performance of smartphone based wide-field retinal imaging (WFI) versus ultra-wide-field imaging (UWFI) for assessment of sight-threatening diabetic retinopathy (STDR) as well as locating predominantly peripheral lesions (PPL) of DR.

**Methods:**

Individuals with type 2 diabetes with varying grades of DR underwent nonmydriatic UWFI with Daytona Plus camera followed by mydriatic WFI with smartphone-based Vistaro camera at a tertiary care diabetes centre in South India in 2021–22. Grading of DR as well as identification of PPL (DR lesions beyond the posterior pole) in the retinal images of both cameras was performed by senior retina specialists. STDR was defined by the presence of severe non-proliferative DR, proliferative DR or diabetic macular oedema (DME). The sensitivity and specificity of smartphone based WFI for detection of PPL and STDR was assessed. Agreement between the graders for both cameras was compared.

**Results:**

Retinal imaging was carried out in 318 eyes of 160 individuals (mean age 54.7 ± 9 years; mean duration of diabetes 16.6 ± 7.9 years). The sensitivity and specificity for detection of STDR by Vistaro camera was 92.7% (95% CI 80.1–98.5) and 96.6% (95% CI 91.5–99.1) respectively and 95.1% (95% CI 83.5–99.4) and 95.7% (95% CI 90.3–98.6) by Daytona Plus respectively. PPL were detected in 89 (27.9%) eyes by WFI by Vistaro camera and in 160 (50.3%) eyes by UWFI. However, this did not translate to any significant difference in the grading of STDR between the two imaging systems. In both devices, PPL were most common in supero-temporal quadrant (34%). The prevalence of PPL increased with increasing severity of DR with both cameras (*p* < 0.001). The kappa comparison between the 2 graders for varying grades of severity of DR was 0.802 (*p* < 0.001) for Vistaro and 0.753 (*p* < 0.001) for Daytona Plus camera.

**Conclusion:**

Mydriatic smartphone-based widefield imaging has high sensitivity and specificity for detecting STDR and can be used to screen for peripheral retinal lesions beyond the posterior pole in individuals with diabetes.

## Introduction

Regular repetitive documentation of retinal lesions by fundus photography is essential for screening as well as management and follow-up of individuals with diabetes/ with and without diabetic retinopathy (DR) [1]. The gold standard for grading of DR in major international studies/ randomised clinical trials has been based on the ETDRS (Early Treatment of Diabetic Retinopathy Study) standard seven-field 30° retinal colour photography that captures DR lesions in the central third of the retina [1]. Nonmydriatic fundus cameras that are used for DR screening cover the central 40–45° of the posterior pole of the retina [2].

While the retinal periphery is affected in a variety of retinal disorders including DR, fundus photography with traditional fundus cameras captures only the central 30–50° of the retina and the lesions in the peripheral retina which are visualised through indirect ophthalmoscopy often remained undocumented by conventional retinal imaging. This has led to the development of wide-field fundus imaging (WFI) systems over the recent years [3]. WFI provides valuable information about the peripheral retinal vasculature and peripheral retinal lesions by imaging beyond 50° field (beyond posterior pole). Ultra-wide field fundus imaging (UWFI) with scanning laser ophthalmoscope (SLO) such as the Optos Daytona or Zeiss Clarus cameras can image from 100-upto 200°, that can cover over 80% of the retinal surface area through a single click compared to 15% retinal surface covered by a single 45° image [4]. UWFI can capture peripheral retinal lesions outside the traditional 7-fields without dilatation. In individuals with diabetes, the Optos camera showed presence of peripheral DR lesions in 1/3rd of eyes [5]. Studies have shown that presence of these peripheral lesions correlate with increased risk of DR progression [6]. Studies have compared and shown good agreement for DR severity assessment between the ETDRS 7-field images and the equivalent area on an UWF image [7–9].

UWF photography is getting considered as the gold standard for DR screening in some of the developed counties like the United States [7]. However, it is an expensive modality for screening DR for regular screening especially for low-and middle-income countries (LMIC) like India [2]. Smartphone-based fundus cameras that are portable and easy to handle in remote places are popular as cost-effective DR screening options [10, 11].

This study utilizes an indigenous, sleek smartphone-based camera for mydriatic WFI [12]. The primary objective of the study was to assess WFI with Remidio Vistaro Camera versus UWFI with Optos Daytona Plus camera with respect to detection of sight-threatening DR (STDR) that requires referral as well as locating peripheral lesions (PPL) of DR. Secondary objectives were to measure the agreement in DR grading of varying severity and analyse intergrader reliability between two senior grader ophthalmologists for both cameras.

## Methods

This was a cross-sectional instrument validation study conducted at the department of ophthalmology of a tertiary care diabetes centre in Chennai, South India. The study was carried out over a period of 6 months (August 2021–Feb 2022) after obtaining approval from the Institutional Ethical Committee of Madras Diabetes Research Foundation, Chennai, India. A written informed consent was obtained from all the participants.

As the purpose of the study was imaging of peripheral DR lesions, consecutive individuals with known diabetes, aged 18 years and above with varying duration of diabetes undergoing regular management and diabetes care at a tertiary care diabetes centre in south India, who had varying grades of DR in the previous retinal examination visit (identified from the electronic medical record) and willing to undergo retinal colour photography for screening of DR through two fundus cameras were invited to participate in the study. Individuals who had undergone panretinal laser photocoagulation treatment or intravitreal injections for treatment of DR were excluded from the study. The sample size was calculated to have a power of >80%; imaging of 294 eyes (147 individuals) was required based on the prevalence of peripheral DR lesions in an earlier study [5].One hundred and sixty individuals were recruited in the study keeping in mind that that about 10% of the images may be ungradable.

### The devices

**Fig. 1:**
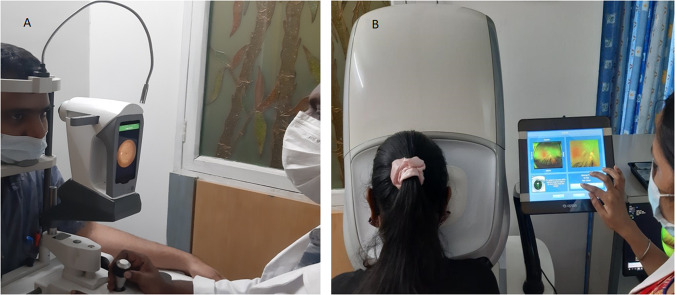
Wide-field retinal imaging in study participants with diabetes. **A** Retinal Imaging with Remidio Vistaro camera. **B** Retinal Imaging with Optos Daytona Plus camera.

Remidio Vistaro (Remidio Innovative Solutions Pvt Ltd, Bangalore, India) is novel, portable smartphone-based, mydriatic, widefield retinal imaging device with autofocus and autocapture capabilities with a 65° field of view (FOV) in a single capture (Fig. 1A) [12]. A montage of 2 images provides a 90° FOV.

Optos Daytona Plus (Optos, Dunfermline, United Kingdom) is a non-contact confocal scanning laser ophthalmoscope (SLO) camera (Fig. 1B) that uses red (633 nm) and green (532 nm) lasers for retinal colour images (pseudocolor) and provides 200° FOV of the retina through an undilated pupil using an ellipsoid mirror and virtual point technology [2, 3].

### Process

After a preliminary eye examination, testing the visual acuity, intraocular pressure measurement and a slit-lamp examination of the anterior segment, Optomap retinal images of both eyes were obtained using the Optos Daytona Plus camera. Then both eyes were dilated and two retinal colour photographs were obtained using Remidio Vistaro camera. A macula-centred image and an optic disc-centred image were captured in each eye and a montage was created. The retinal images were obtained through both cameras by certified trained optometrists following imaging guidelines.

**Fig. 2:**
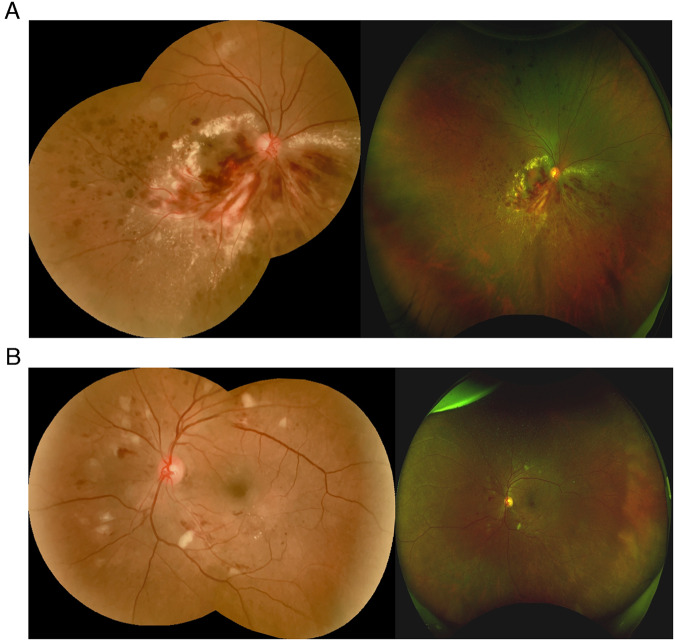
Retinal images taken using the two imaging systems. **A** Right eye: Retinal image of individual with sight-threatening diabetic retinopathy (STDR) with Vistaro and Daytona Plus cameras. **B** Left eye: Retinal image of individual with moderate to severe non-proliferative diabetic retinopathy with Vistaro and Daytona Plus cameras.

Figure 2A and B show the Optomap UWF image and Vistaro WF montaged image of two eyes with various DR lesions.

### Definitions

Wide-field retinal image (WFI) is a retinal photograph which captures retinal anatomic features beyond the posterior pole with a 60–100° FOV [4].

Ultra-widefield retinal image (UWFI) is a single fovea centred retinal photograph that captures retinal anatomic features anterior to the vortex vein ampullae in the four (superior, inferior, nasal and temporal) quadrants with a 100–200° FOV [4].

Predominantly peripheral lesions (PPL) are defined as clinical signs of diabetic retinopathy (DR) like microaneurysms (MA), dot and blot/superficial haemorrhages (H), venous beading, intraretinal microvascular abnormality (IRMA) and new vessel elsewhere (NVE) in eyes with DR with these retinal lesions located outside the ETDRS standard seven-fields.

After the retinal imaging was completed, all the retinal images from both cameras were downloaded and made into folders with dummy ids. The anonymised WFI images of Vistaro and the anonymised UWFI optomap images were graded eye-wise as well as quadrant-wise by 2 certified graders/ senior medical retina specialists (Graders 1 and 2) who were masked to the actual ground truth DR diagnosis. Every week, about 50 folders with anonymised retinal images of both eyes taken with either fundus camera were randomly assigned to Grader 1 and Grader 2 for grading. Then, the next set of anonymised images with dummy ids, were randomly assigned until the images of all study participants taken in both cameras were graded by both Graders 1 and 2. Both graders used their laptops to grade the retinal images of both cameras.

The grading of the presence and severity of DR was performed based on the International Clinical classification of Diabetic Retinopathy (ICDR) severity scale [13]. The ICDR scale provides a classification of five stages of DR (i) no apparent retinopathy-No DR; (ii) mild non-proliferative DR (NPDR) (iii) moderate NPDR (iv) severe NPDR and (v) proliferative DR (PDR). Sight-threatening DR (STDR) was defined by the presence of severe NPDR, PDR and/or DME [14]. The grading of DR as well as peripheral lesions by the 2 retina specialists was compared for both devices. The grading of two-field montage image of Vistaro camera was compared with a single Optomap image. A subset of retinal images from both cameras as well as the images where there was a disagreement in the DR diagnosis between Graders 1 and 2 were graded by a third senior retina specialist. The ground truth for DR grades was the retinal examination report DR diagnosis provided for each eye by the senior retina specialist. Excel sheets with the dummy ids and headings were provided to the graders for providing details regarding the DR lesions, the PPLs, the DR severity grades, etc.

### Statistical analysis

For statistical analysis, all participant-related data were de-identified. Continuous variables were expressed as mean ± standard deviation while categorical variables were presented as proportions. Only the gradable retinal images were included in the analysis. Sensitivity and specificity for varying grades of DR were calculated eye-wise with exact binomials with 95% confidence interval (CI). Following the guidelines by Landis and Koch for kappa statistic (0–0.2: slight agreement; 0.21–0.4: fair agreement; 0.41–0.6: moderate agreement; 0.61–0.8: substantial agreement; 0.81–1.00: almost perfect agreement), the Cohen’s kappa statistic was calculated to evaluate the agreement between ophthalmologist graders. A *p*-value of <0.05 was considered a statistical significance. All statistical analyses were performed using SPSS V25.

## Results

One hundred and sixty individuals (120 [75%] males] with varying grades of severity of DR were included in the study. Retinal imaging was carried out in 318 eyes of 160 individuals with type 2 diabetes. One participant had phthisis bulbi in one eye and another participant had mature cataract in one eye and hence single eye imaging was done in both participants. The mean age of the study participants was 54.7 ± 9 years and the mean duration of diabetes was 16.6 ± 7.9 years.

All the retinal images taken with both devices were gradable by both graders. Table 1 shows DR grading of retinal images of two cameras by the two retina specialist graders. Majority of the grading of both graders matched for the WFI of Vistaro for all grades of DR. Detection of PDR was identical for both cameras for both graders. There were small variations in DR grading only in the mild to moderate NPDR categories. The overall grades of severity of DR/ STDR based on UWFI did not significantly vary from the ground truth indirect ophthalmoscope retinal examination by senior retina specialist. Only in 5 eyes graded by retinal examination as Moderate NPDR, the grade changed to Severe NPDR based on UWFI. Referable DR diagnosed did not vary between ground truth and UWFI based diagnosis.

**Table 1:** Grading of severity of diabetic retinopathy by 2 graders of retinal images by two wide field fundus cameras.

Grader 1		Optos Daytona Plus Camera					
	DR severity	No DR *n*	Mild NPDR *n*	Moderate NPDR *n*	Severe NPDR *n*	PDR *n*	TOTAL *n* (%)
Remidio Vistaro Camera	No DR n	6	1	1	0	0	8 (2.5)
	Mild NPDR n	0	17	3	0	0	20 (6.3)
	Moderate NPDR n	0	1	209	1	1	212 (66.7)
	Severe NPDR n	0	0	0	50	2	52 (16.3)
	PDR n	0	0	0	0	26	26 (8.2)
	TOTAL n (%)	6 (1.9)	19 (6.0)	213 (67.0)	51 (16.0)	29 (9.1)	318 (100)

Grader 2	DR severity	No DR *n* (%)	Mild NPDR *n*	Moderate NPDR *n*	Severe NPDR *n*	PDR *n*	TOTAL *n* (%)
Remidio Vistaro Camera	No DR *n*	7	1	0	0	0	8 (2.5)
	Mild NPDR *n*	0	21	3	1	0	25 (7.9)
	Moderate NPDR *n*	0	2	195	7	1	205 (64.5)
	Severe NPDR *n*	0	0	0	54	2	56 (17.6)
	PDR *n*	0	0	0	0	24	24 (7.5)
	TOTAL *n* (%)	7 (2.2)	24 (7.5)	198 (62.3)	62 (19.5)	27 (8.5)	318 (100)

*NPDR* Non-proliferative diabetic retinopathy, *PDR* Proliferative diabetic retinopathy.

The sensitivity and specificity for detecting varying grades of severity of DR by Vistaro as well as Daytona Plus camera against the ground truth is shown in Table 2. The sensitivity and specificity for STDR detection by Vistaro camera was 92.7% (95% CI (80.1–98.5) and 96.6% (95% CI 91.5–99.1) respectively and the sensitivity and specificity for STDR by Daytona Plus was 95.1% (95% CI 83.5–99.4) and 95.7% (95% CI 90.3–98.6) respectively. The degree of agreement in DR grading between the two graders for both cameras was substantial. The kappa comparison between the 2 graders for varying grades of severity of DR was *k* = 0.802 (*p* < 0.001) for Vistaro camera and *k* = 0.753 (*p* < 0.001) for Daytona Plus camera.

**Table 2:** Sensitivity and specificity analysis for different grades of diabetic retinopathy based on wide field imaging (WFI) and ultra-wide field imaging (UWFI).

Retinal Imaging	DR Category	Sensitivity (95% CI)	Specificity (95% CI)	Kappa comparison between graders
Wide-field imaging (WFI) (VISTARO)	NPDR	99.6 (98.0–100.0)	88.9 (51.8–99.7)	0.802 *p* < 0.001
	PDR	77.8 (57.7–91.4)	99.3 (97.5–99.9)	
	STDR	92.7 (80.1–98.5)	96.6 (91.5–99.1)	
Ultra-wide field imaging (UWFI) (DAYTONA PLUS)	NPDR	98.9 (96.9–99.8)	80.0 (38.4–99.5)	0.753 *p* < 0.001
	PDR	80.0 (61.4–92.3)	99.3 (97.4–99.9)	
	STDR	95.1 (83.5–99.4)	95.7 (90.3–98.6)	

Sight-threatening DR: Defined as Severe NPDR and above without and with diabetic macular oedema.

*NPDR* Non-proliferative diabetic retinopathy, *PDR* Proliferative diabetic retinopathy.

All types of PPL were detected in the periphery by retinal imaging through both cameras. The number of peripheral lesions detected by 2 graders in 318 eyes by both modes of retinal imaging is shown in Table 3. PPL were detected in 89 (27.9%) eyes by Vistaro camera and in 160 (50.3%) eyes by UWFI by Daytona plus camera as the field of view was more in UWFI. Peripheral lesions were most commonly seen in the super-temporal quadrants in both cameras in about one-third of eyes (Supplementary Table 1). PPL were more common in temporal quadrants. Intraretinal haemorrhages (>50%), IRMA and NVE were the most common peripheral lesions seen in this cohort. WFI by Vistaro could detect over 50% of the peripheral lesions detected by UWFI. The distribution of PPL across varying grades of severity of DR is shown in Supplementary Fig. 1. None of the eyes with No-DR had peripheral lesions. The prevalence of PPL increased with increasing severity of DR with both cameras (*p* < 0.001); with >80% of eyes with severe NPDR and PDR had PPL detected on UWFI.

**Table 3:** Detection of predominantly peripheral lesions (PPL) by two graders (318 eyes of 160 individuals).

Grader 1				Grader 2			
Remidio Vistaro Camera	Optos Daytona Plus Camera			Remidio Vistaro Camera	Optos Daytona Plus Camera		
	Peripheral lesions absent	Peripheral lesions present	Total		Peripheral lesions absent	Peripheral lesions present	Total
Peripheral lesions absent	153 (98.1%)^a^	77	230	Peripheral lesions absent	153 (96.8%)^a^	71	224
Peripheral lesions present	3	85 (51.9%)^b^	88	Peripheral lesions present	5	89 (55.6%)^b^	94
Total	156	162	318	TOTAL	158	160	318

^a^Specificity.

^b^Sensitivity.

## Discussion

In this study, we found that smartphone-based widefield imaging has a) high sensitivity and specificity for detecting STDR and b) can detect peripheral DR lesions beyond the posterior pole (PPL) in individuals with diabetes.

While standard 7-field retina imaging is the gold standard, it is more time consuming. A wider field of view of retina covered in lesser time frame would be more appealing to the patient undergoing retinal imaging for DR screening and management. Widefield cameras expanded the field of view of retina from 30°–45° to 60° and have reduced the number of images required for ETDRS grading. The ability to image peripheral retina without dilatation in an efficient manner with least discomfort to the patient has made UWFI attractive and the main mode of choice for retinal imaging in the recent years in ophthalmology practice and being considered for tele-ophthalmology DR screening in the western world [7, 15]. Nonmydriatic UWFI with SLO cameras is a boon in instances where conventional fundus cameras may not provide good quality images in individuals with poor mydriasis due to long-term diabetes.

Studies have shown that PPL defined as DR lesions located outside the standard seven fields, increased the risk of DR progression and PDR development over 4 years by 3.2 and 4.7 times, respectively even after adjusting for gender, duration of diabetes, glycaemic control (HbA1c) and severity of DR at baseline [16]. Previous studies [8, 9, 16, 17] have shown that UWFI with Optos fundus camera is reliable in detecting DR, with good sensitivity and specificity of 73% and 96%, respectively for detecting PDR. In our study, UWFI imaging showed 80% sensitivity and 99.3% specificity for detection of PDR.

In the study comparing mydriatic UWFI and ETDRS photographs, Silva PS et al showed that about one-third of DR lesions like dot and blot haemorrhages, IRMA and NVE were located outside the standard ETDRS fields and the frequency of these PPL varied with severity of DR [5]. In our study, WFI with Vistaro camera could detect PPL in about 30% of the eyes in our study. The montaged WFI in our study provided a 90° FOV and the UWFI provided a 200° FOV of the retina and hence there were proportionately more PPL detected by Daytona Plus camera.

Studies have also shown that the PPL were more frequent in the supero-temporal quadrant, in the temporal fields compared with the nasal fields [5, 8]. In our study also we found that over one-third of the PPL were seen in the supero-temporal quadrant followed by the temporal macula and inferior temporal quadrants. More areas of retinal non-perfusion may possibly be the reason for more PPLs in these quadrants. The study by Cherian J et al showed PPL by UWFI using Daytona plus in over 50% of the eyes [18]. In our study too, PPL was detected in over 50% of the eyes on UWFI.

Price et al. compared the Optomap UWFI with ETDRS standard seven field images and found that 15% of the images received a higher grade of severity of DR on Optos UWFI [19]. In our study, while comparing DR grades on Vistaro WFI and Daytona UWFI we did not find significant change in DR severity grades on Optos images.

The Diabetic Retinopathy Study (DRS) was possibly the first major study that made a montage of the standard 7-field images which could cover up to 75° of the retina [20]. A montaged WFI can create a UWF image. The 2-field montage of Vistaro in our study provided a 90° FOV of the retina. In our study, the kappa agreement between the 2 senior grader retina specialists for various grades of DR was substantial for both cameras.

One of the limitations of UWF cameras like Daytona is the variation of colour of the retina image due to the use of laser of different wavelengths [1, 16]. The senior graders who did the image grading in this study felt that the ‘pseudocolor’ retinal image and lash artefacts made grading of certain peripheral lesions like IRMAs difficult to identify on Daytona Plus camera. They felt that the assessment of DME was better with Vistaro and a bit challenging with the UWF camera possibly due to the lower macular resolution. The limitation of smartphone based Vistaro camera is the requirement of dilatation for WFI [12]. With respect to Vistaro camera, the key to detect peripheral DR lesions is obtaining a good quality montaged image which requires good patient cooperation and fixation stability. The limitations of UWFI include the cost, artefacts from eyelashes, eyelid margin and nose and the peripheral distortion.

The strength of this study is this is possibly the first study globally that has compared detection of PPL by WFI by a smartphone-based camera against UWFI by a SLO camera. Montage of Vistaro 2-field WFI took less time with more retinal area covered than imaging with conventional cameras. The high-resolution image quality with no ungradable images, grading performed by certified senior retina specialists, with over 15 years experience providing accurate grading are additional strengths of this study. This instrument validation study done would enable use of WFI in LMIC in telemedicine for DR screening in individuals with diabetes as well as imaging of other peripheral retinal and choroidal disorders. One of the limitations is that it is a cross sectional study. A longitudinal follow-up assessment of peripheral DR lesions with Vistaro camera would enable assessment of risk of progression /development of STDR.

To conclude, mydriatic smartphone-based WFI is efficient and accurate for documenting peripheral DR lesions beyond the posterior pole to a fair extent and can be considered as a cost-effective alternative to UWFI. With innovative advances like automated grading of peripheral DR lesions, better prediction of progression of DR and appropriate DR management would be possible.

## Summary

### What was known before

- Ultra-wide field fundus imaging (UWFI) with scanning laser ophthalmoscope (SLO) can image from 100-upto 200° covering 80% of the retinal surface.
- UWFI can capture peripheral retinal lesions outside the traditional 7-fields in individuals with diabetes. The Optos camera has shown presence of peripheral diabetic retinopathy (DR) lesions in 1/3rd of eyes. Studies have compared and shown good agreement for DR severity assessment between the ETDRS 7-field images and the equivalent area on an UWF image.

### What this study adds

- Wide-field imaging (WFI) with Vistaro, an indigenous smartphone based camera was assessed for detection of sight-threatening diabetic retinopathy and peripheral DR lesions. The 2 field montage smartphone-based WFI provided a 90° field of view of the retina.
- Peripheral DR lesions beyond the posterior pole were detected in 30% of the eyes with Vistaro camera and the prevalence of predominantly peripheral lesions (PPL) increased with increasing grades of severity of DR. PPL were most common in the temporal quadrants. The device showed high sensitivity and specificity for detecting sight-threatening diabetic retinopathy.
- Smartphone-based wide-field imaging (WFI) is efficient in documenting peripheral DR lesions beyond the posterior pole to a great extent and can be considered as a cost-effective alternative to UWFI in low and middle income countries.

### Supplementary information

Supplemental Figure 1

Supplemental Table 1

Supplemental Figure 1 Legend

## Data Availability

All data relevant to the study are included in the article or uploaded as supplementary information. Additional data are available upon reasonable request.
