# Supplementary figures and images for: Wide-field imaging with smartphone based fundus camera: grading of severity of diabetic retinopathy and locating peripheral lesions in diabetic retinopathy
Source: Eye (Lond). 2024 Jan 31;38(8):1471–6. doi: 10.1038/s41433-024-02928-2 (PMC11126401; doi:10.1038/s41433-024-02928-2)

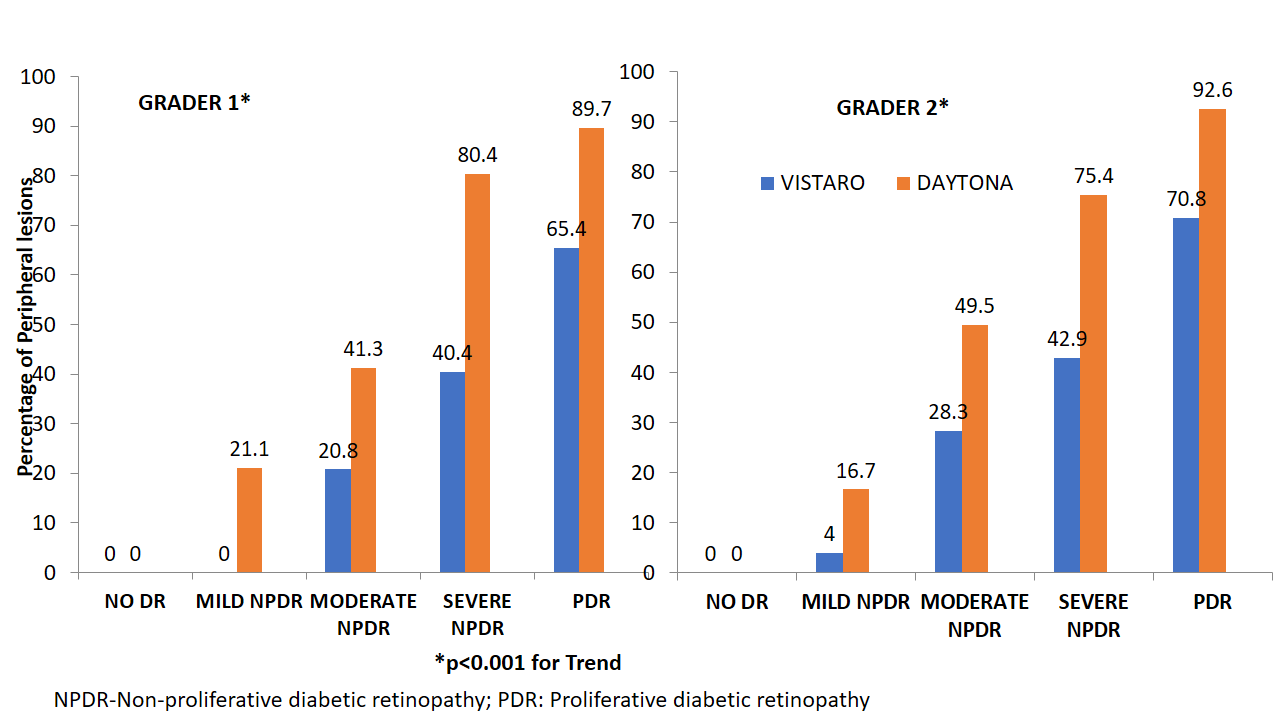

Supplement: Supplementary file 1 — Supplemental Figure 1 [file 41433_2024_2928_MOESM1_ESM.tif]
