# Supplementary material for: Wide-field imaging with smartphone based fundus camera: grading of severity of diabetic retinopathy and locating peripheral lesions in diabetic retinopathy
Source: Eye (Lond). 2024 Jan 31;38(8):1471–6. doi: 10.1038/s41433-024-02928-2 (PMC11126401; doi:10.1038/s41433-024-02928-2)
Supplement: Supplementary file 2 — Supplemental Table 1 [file 41433_2024_2928_MOESM2_ESM.docx]

**Supplementary Table 1: Distribution of predominantly peripheral lesions (PPL) among the five regions in the retina in study participants with diabetic retinopathy**

| **Retinal Quadrants** | **Diabetic Retinopathy Peripheral Lesions in %** | |
| --- | --- | --- |
|  | **Optos Daytona**  **(160 eyes with PPL)** | **Remidio Vistaro**  **(89 eyes with PPL)** |
| **Superior Temporal** | 34.4 | 33.7 |
| **Superior Nasal** | 14.4 | 14.6 |
| **Inferior Nasal** | 10.6 | 9.0 |
| **Inferior Temporal** | 20.6 | 19.1 |
| **Temporal Macula** | 20.0 | 23.6 |
