# Supplementary material for: Wide-field imaging with smartphone based fundus camera: grading of severity of diabetic retinopathy and locating peripheral lesions in diabetic retinopathy
Source: Eye (Lond). 2024 Jan 31;38(8):1471–6. doi: 10.1038/s41433-024-02928-2 (PMC11126401; doi:10.1038/s41433-024-02928-2)
Supplement: Supplementary file 3 — Supplemental Figure 1 Legend [file 41433_2024_2928_MOESM3_ESM.docx]

**Supplementary Figure 1**

The distribution of predominantly peripheral lesions (PPL) across varying grades of severity of diabetic retinopathy
